# Supplementary material for: Interpretable deep learning methods for multiview learning
Source: BMC Bioinformatics. 2024 Feb 14;25:69. doi: 10.1186/s12859-024-05679-9 (PMC11265116; doi:10.1186/s12859-024-05679-9)
Supplement: Supplementary file 1 — Additional file 1. contains information on our optimization and algorithm, hyper-parameter selection and linear simulations results. [file 12859_2024_5679_MOESM1_ESM.pdf]

Supplementary Materials of Interpretable Deep Learning Methods for Multiview Learning

Hengkang Wang[1], Han Lu[2], Ju Sun[1], Sandra E Safo[2*]

[1]Department of Computer Science and Engineering, University of Minnesota, Minneapolis, 55455, USA.
[2*]Division of Biostatistics, University of Minnesota, Minneapolis, 55455, USA.

*Corresponding author(s). E-mail(s): ssafo@umn.edu;
Contributing authors: wang9881@umn.edu; lu000054@umn.edu; jusun@umn.edu;

1 More on Methods

1.1 Optimization and Algorithm

We solve the optimization problems iteratively using ADAM[1] with $\beta_1 = 0.9$ and $\beta_2 = 0.999$. We initialize the weights and biases of the network by using the default settings in PyTorch and the shared low-dimensional representation matrix with numbers drawn from the standard distribution. Our algorithm is divided into three stages. The first stage is the Feature Selection stage. In this stage, we solve the optimization problem (3) or (5) [main text] to obtain features that are highly-ranked. In particular, we feed forward the network with the weights and biases to obtain $G_d(\mathbf{Z})$. We compare $G_d(\mathbf{Z})$ with the training data and measure the error using the loss function. We perform a backwards pass and propagate the error to each individual node using backpropagation. We compute gradients of the loss function with respect to the inputs of iDeepViewLearn (i.e. with respect to the weights and biases), for $\mathbf{Z}$ fixed, and adjust weights using gradient descent. Then, we optimize the loss function with respect to $\mathbf{Z}$, while keeping the weights and biases fixed. We repeat the process iteratively until the difference between the current and previous iteration losses is very small or a maximum number of iterations reached. Please refer to Algorithm 1 for more details.

The second stage is the Reconstruction and Training stage using selected features. Here, we solve the optimization problem (6) [main text]. Our input data are

the observed data with the selected features (i.e. top r or $r\%$ features in each view), $\mathbf{X}'^{(1)} \ldots \mathbf{X}'^{(D)}$. We proceed similarly like the first stage, and we iteratively solve the optimization problem until convergence or some maximum number of iteration reached. At convergence, we obtain the reconstructed data $R_d(\mathbf{Z}')$, and the learned shared low-dimensional representations, $\widetilde{\mathbf{Z}}'$ based on only the top r or $r\%$ variables in each view. Downstream analyses such classification, regression, or clustering could be carried out on this learned shared low-dimensional representations. For instance, for our simulations and real data analyses, we trained a support vector machine classifier using these shared low-dimensional representations. Additionally, for the real data analyses, we trained a K-means clustering algorithm on the shared low-dimensional representation to obtain clusters common to all the views. We note that downstream analyses such as clustering could be implemented on the reconstructed views for view-specific clusters, in addition to the joint clusters. Please refer to Algorithm 2 for more details.

The third stage is the Prediction stage, if an outcome is available. Here, we solve the optimization problem (8) for the learned shared-low dimensional representation ($\widetilde{\mathbf{Z}}'_{test}$) corresponding to the testing views ($\mathbf{X}'^{(1)}_{test} \ldots \mathbf{X}'^{(D)}_{test}$). We use the learned weights and biases from the second stage, and we implement a stochastic gradient descent algorithm. Since the network parameters (i.e. weights and biases) are fixed, we only update the loss function with respect to $\mathbf{Z}'_{test}$. Please refer to Algorithm 3 for more details. We use the automatic differentiation function "autograd" in Pytorch to estimate the gradients of our loss function with respect to the network parameters or latent code. We use Exponential Linear Unit (ELU) [2] as our nonlinear activation function.

1.2 Hyper-Parameter Selection

There are several crucial hyper-parameters for our proposed model, including λ^d, λ'^d, number of latent components K, learning rates of neural networks for various views, and learning rate of the latent code $\mathbf{Z}$. We also add essential hyper-parameters of downstream models to our search space. For example, we also tune regularization parameter C and kernel coefficient γ when adopting Support Vector Machines (SVMs) with Radial Basis Function (RBF) for classification tasks. Particularly, λ^d is set to be 0.1 as default due to its insensitivity; the upper bound of K is r or the minimum of the numbers of features from all the views times $r\%$; learning rates for different neural networks are unified for simplicity. Since the hyper-parameter search space is not small, we obtain all combinations of our hyper-parameters, and we randomly select [3] some combinations of hyper-parameters to search over.

2 More on Simulations

2.1 Linear simulations when prior information is not available

We simulate data with $D = 2$ views, and $K = 3$ classes following the simulation setup in [4]. For each view, we concatenate data from the three classes i.e., $\mathbf{X}^{(d)} = [\mathbf{X}_1^{(d)}, \mathbf{X}_2^{(d)}, \mathbf{X}_3^{(d)}], d = 1, 2$. The combined data $\left(\mathbf{X}_k^{(1)}, \mathbf{X}_k^{(2)}\right)$ for each class are

Algorithm 1 First stage: Feature Selection

Input: Training data from different views $\mathbf{X}^{(1)}, \mathbf{X}^{(2)}, ..., \mathbf{X}^{(D)}$. Each variable in a view is standardized to have mean zero and variance one, but no standardization is allowed when facing images because variables, i.e., pixels are not independent; neural networks $G_1, G_2, ..., G_D$ for each view; Laplacian ($\mathcal{L}^{(d)}$) for network-based approach; learning rate l_1, l_2, ..., l_D for neural networks G_1, G_2, ..., G_D respectively; latent code (or shared low-dimensional representation) $\mathbf{Z}$ with K components; learning rate l_Z for the latent code $\mathbf{Z}$; number of iterations I

Output: Optimized weights and biases for neural networks $G_1, G_2, ..., G_D$; optimized latent code $\mathbf{Z}$; indices $\mathbf{I}_1$, $\mathbf{I}_2$, ...,$\mathbf{I}_D$ for important features of each view

Initialization Initialize neural networks $G_1, G_2, ..., G_D$ by the default settings of *PyTorch*; Assign random numbers from the standard normal distribution for the latent code $\mathbf{Z}$
while $i = 1, 2, ..., I$ **do**
    Feed forward the network of latest weights and biases to obtain the reconstructions of each view (i.e. $G_d(\mathbf{Z})$)
    Apply equation (3) or (5) to calculate losses with $\mathbf{X}^{(1)}, \ldots, \mathbf{X}^{(D)}$
    Compute the gradient of weights and biases for each network and the latend code $\mathbf{Z}$ by the *PyTorch Autograd* function
    Update the weights and biases with specified learning rate α
end while
Feature Selection Feed the learned latent code $\mathbf{Z}$ into neural networks G_1, G_2, ..., G_D to obtain reconstructed data $G_d(\mathbf{Z})$ for the observed data $\mathbf{X}^{(d)}$. Calculate column-wise norm of $G_d(\mathbf{Z})$. Choose the columns with large column norm as important features for that view. Save the indices of important features as $\mathbf{I}_1$, $\mathbf{I}_2$, ...,$\mathbf{I}_D$. Denote the observed datasets with only the selected features as $\mathbf{X}'^{(1)}, \mathbf{X}'^{(2)}, ..., \mathbf{X}'^{(D)}$.

simulated from $N(\boldsymbol{\mu}_k, \boldsymbol{\Sigma})$, where $\boldsymbol{\mu}_k = (\boldsymbol{\mu}_k^{(1)}, \boldsymbol{\mu}_k^{(2)})^{\mathrm{T}} \in \mathbb{R}^{p^{(1)}+p^{(2)}}, k = 1, 2, 3$ is the combined mean vector for class k; $\boldsymbol{\mu}_k^{(1)} \in \mathbb{R}^{p^{(1)}}$, $\boldsymbol{\mu}_k^{(2)} \in \mathbb{R}^{p^{(2)}}$ are the mean vectors for $\mathbf{X}_k^{(1)}$ and $\mathbf{X}_k^{(2)}$ respectively. The true covariance matrix $\boldsymbol{\Sigma}$ is partitioned as

$$\boldsymbol{\Sigma} = \begin{pmatrix} \boldsymbol{\Sigma}^1 & \boldsymbol{\Sigma}^{12} \\ \boldsymbol{\Sigma}^{21} & \boldsymbol{\Sigma}^2 \end{pmatrix}, \boldsymbol{\Sigma}^1 = \begin{pmatrix} \widetilde{\boldsymbol{\Sigma}}^1 & \mathbf{0} \\ \mathbf{0} & \mathbf{I}_{p-20} \end{pmatrix}, \boldsymbol{\Sigma}^2 = \begin{pmatrix} \widetilde{\boldsymbol{\Sigma}}^2 & \mathbf{0} \\ \mathbf{0} & \mathbf{I}_{q-20} \end{pmatrix}$$

where $\boldsymbol{\Sigma}^1$, $\boldsymbol{\Sigma}^2$ are, respectively, the covariance of $\mathbf{X}^{(1)}$ and $\mathbf{X}^{(2)}$, and $\boldsymbol{\Sigma}^{12}$ is the cross-covariance between the two views. $\widetilde{\boldsymbol{\Sigma}}^1$ and $\widetilde{\boldsymbol{\Sigma}}^2$ are each block diagonal with 2 blocks of size 10, between block correlation 0, and each block is a compound symmetric matrix with correlation 0.8. We generate $\boldsymbol{\Sigma}^{12}$ as follows. Let $\mathbf{V}^1 = [\mathbf{V}_1^1,\ \mathbf{0}_{(p^{(1)}-20)\times 2}]^{\mathrm{T}} \in \mathbb{R}^{p^{(1)}\times 2}$ where the entries of $V_1^1 \in \mathbb{R}^{20\times 2}$ are *i.i.d* samples from U(0.5,1). We similarly define $\mathbf{V}^2$ for the second view, and normalize such that $\mathbf{V}^{1^T}\boldsymbol{\Sigma}^1\mathbf{V}^1 = \mathbf{I}$ and $\mathbf{V}^{2^{\mathrm{T}}}\boldsymbol{\Sigma}^2\mathbf{V}^2 = \mathbf{I}$. We then set $\boldsymbol{\Sigma}^{12} = \boldsymbol{\Sigma}^1\mathbf{V}^1\mathbf{D}\mathbf{V}^{2^{\mathrm{T}}}\boldsymbol{\Sigma}^2$, $\mathbf{D} = \mathrm{diag}(\rho_1, \rho_2)$ to represent a moderate association between the views. For the separation between classes, we take

Algorithm 2 Second stage: Reconstruction and Training

Input: Training data from different view $\mathbf{X}^{(1)}, \mathbf{X}^{(2)}, ..., \mathbf{X}^{(D)}$, optional class labels or outcome $\mathbf{y}$; neural networks $R_1, R_2, ..., R_D$ for each view; number of components K for latent code $\mathbf{Z}'$; learning rate l'_1, l'_2, ..., l'_D for neural networks R_1, R_2, ..., R_D respectively; learning rate l'_Z for the latent code $\mathbf{Z}'$; number of iterations I; indices $\mathbf{I}_1$, $\mathbf{I}_2$, ...,$\mathbf{I}_D$ for selected features for views $1, \ldots, D$, respectively.

Output: Optimized weights and biases for neural networks $R_1, R_2, ..., R_D$; Optimized latent code $\mathbf{Z}'$ (denoted as $\widetilde{\mathbf{Z}}'$); a trained classifier $\mathbf{C}$ or prediction model

Initialization Initialize neural networks $R_1, R_2, ..., R_D$ by the default settings of *PyTorch*; Assign random numbers from the standard normal distribution for the latent code $\mathbf{Z}'$; Obtain $\mathbf{X}'^{(1)}, \mathbf{X}'^{(2)}, ..., \mathbf{X}'^{(D)}$ by applying feature indices $\mathbf{I}_1$, $\mathbf{I}_2$, ...,$\mathbf{I}_D$ to training data

while $i = 1, 2, ..., I$ **do**

Feed forward the network of latest weights and biases to obtain the reconstructions of each view (i.e. $R_d(\mathbf{Z}')$)

Apply equation (7) to calculate losses with $\mathbf{X}'^{(1)}, \mathbf{X}'^{(2)}, ..., \mathbf{X}'^{(D)}$

Compute the gradient of weights and biases for each network and the latent code Z by the *PyTorch Autograd* function

Update the weights and biases with specified learning rate α

end while

Training Train a classifier $\mathbf{C}$ by using the trained latent code $\widetilde{\mathbf{Z}}'$ and outcome $\mathbf{y}$

Algorithm 3 Last stage: Testing

Input: Testing data from different view $\mathbf{X}_{test}^{(1)}, \mathbf{X}_{test}^{(2)}, ..., \mathbf{X}_{test}^{(D)}$; learned neural networks $R_1, R_2, ..., R_D$ (i.e. weights and biases) for each view; number of components K for latent code $\mathbf{Z}'_{test}$; learning rate l_{test} for the latent code $\mathbf{Z}'_{test}$; number of iterations I; indexes $\mathbf{I}_1$, $\mathbf{I}_2$, ...,$\mathbf{I}_D$ for selected features for views $1, \ldots, D$, respectively; (optional) trained classifier ($\mathbf{C}$).

Output: Optimized latent code $\mathbf{Z}'_{test}$ of testing data (denoted as $\widetilde{\mathbf{Z}}'_{test}$); predicted outcome $\widehat{\mathbf{y}}_{test}$

Initialization Fix neural networks $R_1, R_2, ..., R_D$ trained in the second stage; Assign random numbers to latent code $\mathbf{Z}_{test}$; Obtain $\mathbf{X}'^{(1)}_{test}, \mathbf{X}'^{(2)}_{test}, ..., \mathbf{X}'^{(D)}_{test}$ by applying important features $\mathbf{I}_1$, $\mathbf{I}_2$, ...,$\mathbf{I}_D$ to testing data

while $i = 1, 2, ..., I$ **do**

Feed forward the network by the latest $\mathbf{Z}_{test}$ to obtain the reconstructions of each view (i.e $R_d(\mathbf{Z}'_{test})$)

Apply equation (8) to calculate losses with $\mathbf{X}'^{(1)}_{test}, \mathbf{X}'^{(2)}_{test}, ..., \mathbf{X}'^{(D)}_{test}$

Update $\mathbf{Z}'_{test}$ with specified learning rate l_{test}

end while

Testing Put learned $\widetilde{\mathbf{Z}}'_{test}$ into the trained classifier $\mathbf{C}$ and obtain the predicted outcome $\widehat{\mathbf{y}}_{test}$

$\boldsymbol{\mu}_k$ as the columns of $[\boldsymbol{\Sigma}\mathbf{A}, \mathbf{0}_{p^{(1)}+p^{(2)}}]$, and $\mathbf{A} = [\mathbf{A}^1, \mathbf{A}^2]^{\mathrm{T}} \in \mathrm{Re}^{(p^{(1)}+p^{(2)})\times 2}$. Here, the first column of $\mathbf{A}^1 \in \mathbb{R}^{p^{(1)}\times 2}$ is set to $(c_1\mathbf{1}_{10}, \mathbf{0}_{p^{(1)}-10})$ and the second column is set to $(\mathbf{0}_{10}, -c\mathbf{1}_{10}, \mathbf{0}_{p^{(1)}-20})$. We set $\mathbf{A}^2 \in \mathbb{R}^{p^{(2)}\times 2}$ similarly. We consider different combinations of (ρ_1, ρ_2, c). For each combination, we consider the equal class size $n_k = 180$ and dimensions $(p^{(1)}/p^{(2)} = 1000/1000)$. The true number of signal variables is 20.

The proposed method was implemented in the training data, and we identified the top 20 variables. We learned a new model with only the top 20 variables and used the learned model and the testing data to obtain the test error. We report the results of our method and the competitors in Table S1. Compared to the nonlinear association-based method, Deep CCA, the proposed method achieved lower misclassification rates across all settings. The proposed method had comparable error rates in Settings 1 and 3, and lower error rate in Setting 2 compared to the linear association-based method. Compared to SVM in stacked data, the proposed method had lower average error rates across all settings. Since Deep CCA is not capable of variable selection, we coupled Deep CCA with the TS network. The performance of the proposed method in identifying the 20 signal variables was comparable with Sparse CCA and better than Deep CCA + TS. Furthermore, the proposed method had zero false positives, suggesting that the method was capable of not falsely ranking the noise variables in the top 20. The TS framework for ranking variables was suboptimal, as is evident from the TPR, FPR, and F measures for Deep CCA + TS. Random forest has very comparable classification and feature selection performance with our method in the simple linear simulations. The detailed network structures of linear and nonlinear settings are shown in Table S4, respectively.

3 More on Experiment Settings

In this section, we present detailed hyper-parameter settings for our method and all competing methods to enhance reproducibility. The hyper-paramaters of our proposed method, MOMA (+ SVM), Deep CCA + SVM, Sparse CCA + SVM, SVM and random forest are shown in Tables S2 to S7, respectively. The hyper-parameters of the methods that are trained with default parameters are listed in Table S8.

Tab. S1 Linear Settings: randomly select combinations of hyper-parameters to search over. TPR-1; true positive rate for $\mathbf{X}^{(1)}$. Similar for TPR-2. FPR; false positive rate for $\mathbf{X}^{(2)}$. Similar for FPR-2; F-1 is the F measure for $\mathbf{X}^{(1)}$. Similar for F-2. The highest F-1/2 is in red. (The mean error of two views is reported for MOMA; MOMA + SVM means combining the feature selection part of MOMA and SVM.)

Method	Error (%)	TPR-1	TPR-2	FPR-1	FPR-2	F-1	F-2
**Setting 1**							
($\rho_1 = 0.9, \rho_2 = 0.7, c = 0.5$)							
**iDeepViewLearn**	0.09 (0.07)	100.00	100.00	0.00	0.00	100.00	100.00
iDeepViewLearn on stacked data	0.09 (0.09)	100.00	100.00	0.00	0.00	100.00	100.00
Sparse CCA + SVM	0.10 (0.06)	100.00	100.00	0.42	0.25	91.67	94.95
Deep CCA + TS + SVM	6.53 (1.77)	3.50	2.25	1.97	1.99	3.50	2.25
MOMA	25.18 (5.61)	86.50	86.25	0.28	0.28	86.50	86.25
MOMA + SVM	0.04 (0.07)	86.50	86.25	0.28	0.28	86.50	86.25
Random Forest on stacked data	0.02 (0.04)	100.00	100.00	0.00	0.00	100.00	100.00
SVM on stacked data	0.16 (0.15)	-	-	-	-	-	-
**Setting 2**							
($\rho_1 = 0.15, \rho_2 = 0.05, c = 0.12$)							
**iDeepViewLearn**	31.61 (1.21)	100.00	100.00	0.00	0.00	100.00	100.00
iDeepViewLearn on stacked data	32.04 (1.40)	100.00	100.00	0.00	0.00	100.00	100.00
Sparse CCA + SVM	38.60 (3.48)	89.50	90.25	0.01	0.01	91.51	93.40
Deep CCA + TS + SVM	45.09 (2.02)	2.50	2.25	1.99	1.99	2.50	2.25
MOMA	43.15 (2.49)	67.25	67.75	0.67	0.66	67.25	67.75
MOMA + SVM	31.25 (2.30)	67.25	67.75	0.67	0.66	67.25	67.75
Random Forest on stacked data	31.20 (1.85)	99.75	100.00	0.01	0.00	99.75	100.00
SVM on stacked data	32.57 (1.89)	-	-	-	-	-	-
**Setting 3**							
($\rho_1 = 0.9, \rho_2 = 0.7, c = 0.5$)							
**iDeepViewLearn**	0.01 (0.03)	100.00	100.00	0.00	0.00	100.00	100.00
iDeepViewLearn on stacked data	0.04 (0.10)	100.00	100.00	0.00	0.00	100.00	100.00
Sparse CCA + SVM	0.00 (0.00)	100.00	100.00	0.31	0.24	93.71	94.83
Deep CCA + TS + SVM	5.63 (1.96)	3.00	2.50	1.89	1.90	3.00	2.50
MOMA	19.26 (5.90)	94.25	89.00	0.12	0.22	94.25	89.00
MOMA + SVM	0.00 (0.00)	94.25	89.00	0.12	0.22	94.25	89.00
Random Forest on stacked data	0.00 (0.00)	100.00	100.00	0.00	0.00	100.00	100.00
SVM on stacked data	0.03 (0.07)	-	-	-	-	-	-

Tab. S2 iDeepViewLearn.

Data	K	lr	lrz	C	Gamma
Linear Setting 1	16	0.0001	1	1	scale
Linear Setting 2	12	0.0001	1	0.1	scale
Linear Setting 3	12	0.0001	1	100	scale
Nonlinear Setting 1	10	0.0001	1	1	scale
Nonlinear Setting 2	10	0.0001	1	0.1	scale
Nonlinear Setting 3	40	0.0001	1	10	scale
Scale-free Setting 1	12	0.0001	10	1	scale
Scale-free Setting 2	16	0.1	1	1	scale
Lattice Setting 1	9	0.0001	1	10	scale
Lattice Setting 2	9	0.0001	1	10	scale
Cluster Setting 1	6	0.0001	10	1	scale
Cluster Setting 2	6	0.1	1	10	scale
Holm Breast Cancer Study (top 10%)	30	0.1	1	0.1	scale
Holm Breast Cancer Study (top 20%)	30	0	0.1	1	scale
LGG (top 50)	30	0.1	1	1	scale
LGG (top 100)	40	0.001	10	1	scale

Tab. S3 MOMA and MOMA + SVM.

Data	Number of modules	Weight decay	Patience number	C	Gamma
Linear Setting 1	64	0.001	50	10	scale
Linear Setting 2	128	0.001	100	0.1	scale
Linear Setting 3	128	0.001	100	0.1	scale
Nonlinear Setting 1	32	0.00	100	10	scale
Nonlinear Setting 2	128	0.001	50	1	scale
Nonlinear Setting 3	32	0.00	50	1	scale
Scale-free Setting 1	128	0.00	100	1	scale
Scale-free Setting 2	64	0.001	50	1	scale
Lattice Setting 1	128	0.00	100	1	scale
Lattice Setting 2	32	0.00	50	1	scale
Cluster Setting 1	64	0.001	100	1	scale
Cluster Setting 2	128	0.00	50	10	scale
Holm Breast Cancer Study	64	0.001	100	1	scale

Tab. S4 Deep CCA+SVM, all with full batch for DCCA and rbf kernel for SVM.

Data	Network Structure	Epochs per run	C	Gamma
Linear Setting 1	Input-256*10-64-20	50	0.1	scale
Linear Setting 2	Input-256*10-64-20	50	1	scale
Linear Setting 3	Input-256*10-64-20	50	0.1	scale
Nonlinear Setting 1	Input-256*10-64-20	50	1	scale
Nonlinear Setting 2	Input-256*10-64-20	50	10	scale
Nonlinear Setting 3	Input-256*10-64-20	50	1	0.1
Scale-free Setting 1	Input-256*10-64-20	50	1	scale
Scale-free Setting 2	Input-256*10-64-20	50	1	scale
Lattice Setting 1	Input-256*10-64-20	50	1	scale
Lattice Setting 2	Input-256*10-64-20	50	1	scale
Cluster Setting 1	Input-256*10-64-20	50	1	scale
Cluster Setting 2	Input-256*10-64-20	50	1	scale
Holm Breast Cancer Study	Input-256*10-64-20	50	1	scale
MNIST	Input-256*10-64-20	50	10	scale

Tab. S5 Sparce CCA+SVM, all with ncancorr = 1, CovStructure = 'Iden', nfolds = 5, ngrid = 10, standardize = TRUE, thresh = 0.0001, and maxiteration = 20 for SCCA and all with rbf kernel for SVM.

Data	C	Gamma
Linear Setting 1	0.1	scale
Linear Setting 2	0.1	scale
Linear Setting 3	1	scale
Nonlinear Setting 1	10	0.1
Nonlinear Setting 2	1	1
Nonlinear Setting 3	10	0.1
Scale-free Setting 1	1	scale
Scale-free Setting 2	1	scale
Lattice Setting 1	1	scale
Lattice Setting 2	1	scale
Cluster Setting 1	1	scale
Cluster Setting 2	1	scale
Holm Breast Cancer Study	1	scale

Tab. S6 SVM on stacked data, all with rbf kernel.

Data	C	Gamma
Linear Setting 1	10	scale
Linear Setting 2	1	scale
Linear Setting 3	0.1	scale
Nonlinear Setting 1	1	scale
Nonlinear Setting 2	10	scale
Nonlinear Setting 3	1	scale
Scale-free Setting 1	1	scale
Scale-free Setting 2	1	scale
Lattice Setting 1	1	scale
Lattice Setting 2	1	scale
Cluster Setting 1	1	scale
Cluster Setting 2	1	scale
Holm Breast Cancer Study	1	scale
MNIST	1	scale
LGG	1	scale

Tab. S7 Random forest.

Data	n_estimators	max_depth	min_samples_split	min_samples_leaf
Linear Setting 1	200	20	5	2
Linear Setting 2	200	None	5	4
Linear Setting 3	200	None	5	1
Nonlinear Setting 1	100	None	5	1
Nonlinear Setting 2	200	30	2	1
Nonlinear Setting 3	200	None	10	1
Scale-free Setting 1	200	30	2	1
Scale-free Setting 2	200	20	5	2
Lattice Setting 1	200	None	5	1
Lattice Setting 2	200	None	10	2
Cluster Setting 1	50	20	10	4
Cluster Setting 2	200	30	2	1
Holm Breast Cancer Study	50	30	5	1
LGG	100	20	5	4

Tab. S8 Other methods that are trained with their default hyper-parameters.

Method	Analysis	Default hyper-parameters
Fused CCA	Simulation with variable-variable connections	method='Fused',constopt='OptB', mygamma=2, myeta=0.5, nfolds=5, ngrid=10, thresh=1e-04, asnormalize=TRUE, maxiteration=20 and SVM with rbf kernel, C=1, gamma='scale'
SIDA	LGG	gridMethod='RandomSearch', AssignClassMethod='Joint', nfolds=5, ngrid=8, standardize=TRUE, maxiteration=20, weight=0.5, thresh=1e-03
Deep IDA	LGG	network structure=input-256*10-64-20, learning rate=0.01, epoch=30
DGCCA + SVM	LGG	network structure=input-128-128-20, epochs=400, C=1, gamma='scale', weight decay=0.0001

References

[1] Kingma, D.P., Ba, J.: Adam: A Method for Stochastic Optimization. arXiv. Number: arXiv:1412.6980 arXiv:1412.6980 [cs] (2017). http://arxiv.org/abs/1412.6980 Accessed 2022-06-02

[2] Clevert, D.-A., Unterthiner, T., Hochreiter, S.: Fast and Accurate Deep Network Learning by Exponential Linear Units (ELUs). arXiv. Number: arXiv:1511.07289 arXiv:1511.07289 [cs] (2016). http://arxiv.org/abs/1511.07289 Accessed 2022-05-26

[3] Bergstra, J., Bengio, Y.: Random search for hyper-parameter optimization. J. Mach. Learn. Res. **13**, 281–305 (2012)

[4] Safo, S.E., Min, E.J., Haine, L.: Sparse linear discriminant analysis for multiview structured data. Biometrics **n/a**(n/a) (2021) https://doi.org/10.1111/biom.13458 https://onlinelibrary.wiley.com/doi/pdf/10.1111/biom.13458
